# Void space inside the developing seed of *Brassica napus* and the modelling of its function

**DOI:** 10.1111/nph.12342

**Published:** 2013-05-21

**Authors:** Pieter Verboven, Els Herremans, Ljudmilla Borisjuk, Lukas Helfen, Quang Tri Ho, Henning Tschiersch, Johannes Fuchs, Bart M Nicolaï, Hardy Rolletschek

**Affiliations:** 1 BIOSYST- MeBioS, Faculty of Bioscience Engineering, University of LeuvenW. de Croylaan 42, 3001, Leuven, Belgium; 2 Leibniz-Institute of Plant Genetics and Crop Plant Research (IPK)Corrensstrasse 3, 06466, Gatersleben, Germany; 3 IPS/ANKA, Karlsruhe Institute of TechnologyPO Box 3640, 76021, Karlsruhe, Germany; 4 ESRF6 rue Jules Horowitz, BP220, 38043, Grenoble Cedex, France

**Keywords:** *Brassica napus*, gas exchange, mitochondrial respiration, oxygen diffusivity, synchrotron X-ray computed tomography

## Abstract

- The developing seed essentially relies on external oxygen to fuel aerobic respiration, but it is currently unknown how oxygen diffuses into and within the seed, which structural pathways are used and what finally limits gas exchange.
- By applying synchrotron X-ray computed tomography to developing oilseed rape seeds we uncovered void spaces, and analysed their three-dimensional assembly. Both the testa and the hypocotyl are well endowed with void space, but in the cotyledons, spaces were small and poorly inter-connected. In silico modelling revealed a three orders of magnitude range in oxygen diffusivity from tissue to tissue, and identified major barriers to gas exchange.
- The oxygen pool stored in the voids is consumed about once per minute. The function of the void space was related to the tissue-specific distribution of storage oils, storage protein and starch, as well as oxygen, water, sugars, amino acids and the level of respiratory activity, analysed using a combination of magnetic resonance imaging, specific oxygen sensors, laser micro-dissection, biochemical and histological methods.
- We conclude that the size and inter-connectivity of void spaces are major determinants of gas exchange potential, and locally affect the respiratory activity of a developing seed.

## Introduction

Given that the seed and/or the fruit is the key product of most crop species, their development has been intensively investigated (Moles *et al*., 2005; Weber *et al*., 2005; De Smet *et al*., 2010; North *et al*., 2010). Fertilization is followed by an active period of cell division, during which the maternal and filial tissues develop in a coordinated fashion. The filial embryo and endosperm are enclosed by the maternal integument, which eventually forms the testa. Within the seed, cell expansion is accompanied by the accumulation of storage products (proteins, starch and oils). As long as oxygen is available, the metabolism of the developing seed can derive its energy from aerobic respiration. Both seed number and size are dependent on the atmospheric oxygen level, as shown for a number of plant species (Akita & Tanaka, 1973; Quebedeaux & Hardy, 1975; Musgrave & Strain, 1988; Kuang *et al*., 1998), indicating that steep concentration gradients are required to deliver oxygen into the inner portion of the seed. Where oxygen influx falls below the demand, development becomes distorted or is even aborted. The assessment of the endogenous oxygen level within the seed has demonstrated that oxygen shortage (hypoxia) occurs regularly during the course of development (Borisjuk & Rolletschek, 2009). While much work has been devoted towards the implications of hypoxia for seed physiology and crop yield, as yet neither the pathway whereby oxygen diffuses into the seed, nor the diffusivity of seed tissue and the identity of the major diffusional barriers, nor the extent to which these are genetically and/or environmentally influenced are currently understood. Quantitative phase contrast X-ray tomography studies on mature (dry) *Arabidopsis thaliana* seeds (Cloetens *et al*., 2006) identified a network of channels, thought to represent perhaps an oxygen storage space for use during germination, or perhaps a water or gas transport system. As O_2_ diffusion in air is *c*. 10 000 times faster than in water, gas-filled, interconnected pores could determine the gas exchange potential of the developing seed, and thus affect respiratory activity, storage metabolism and seed growth. An important issue that remains is whether these networks are gas- or water-filled during seed development, and how this affects the oxygen diffusivity/transport capabilities of the developing seed. The functional significance of similar void networks in certain fruits has been addressed by determining the void volume, and characterizing the diffusivity of oxygen facilitated by them (Ho *et al*., 2011).

The aim of the present study was to ascertain whether the developing seed forms a void network, and if so, the extent to which it is responsible for oxygen transport or storage. The platform used to achieve this aim was X-ray micro-computed tomography (CT), using both microfocus X-ray sources and synchrotron radiation. As CT is a non-invasive procedure, it avoids the many artefacts that inevitably arise when tissue is cut and fixed. Therefore it allows for the *in vivo* visualization of cellular architecture at a micrometer level of resolution (Cloetens *et al*., 2006; Verboven *et al*., 2008; Lombi & Susini, 2009). The CT data collected enabled a reconstruction of the three-dimensional distribution of pore spaces in the developing seed, an estimation of tissue-specific porosity and diffusivity, and the modelling of oxygen distribution, storage and exchange. In addition we assessed the spatial distribution of storage products, respiration, oxygen, water, sugars and amino acids using a combination of magnetic resonance imaging (MRI), specific oxygen sensors, laser micro-dissection, and established biochemical and histological methods. The focus of investigation was oilseed rape (*Brassica napus*), which is both a close relative of the model plant *A. thaliana*, and a significant commercial source of plant lipids (Biermann *et al*., 2011). We here demonstrate that the airspaces in developing *B. napus* seeds are small and sparsely interconnected in cotyledons, but allow high gas exchange rates in both the seed coat and along the axial direction of the hypocotyl. Implications for gaseous diffusion and respiratory control are discussed.

## Materials and Methods

### Plant material and growth

Plants of oilseed rape (*Brassica napus* L. var. Miniraps) were grown in a phytochamber at 18°C with 16 h of light (400 μmol quanta m^−2^ s^−1^) and a relative air humidity of 60%. At the time of flowering, plants were tagged for determination of developmental stages. Seeds were isolated at mid storage stage (*c*. 30 d after fertilization) and used for subsequent analysis.

### Micro-CT of whole rapeseeds and tissues

Whole rapeseeds were scanned using a SkyScan 1172 high resolution X-ray micro-CT system (SkyScan, Kontich, Belgium), operating at 59 keV, a rather low X-ray energy that is well suited for scanning soft, biological materials. Experimental conditions were optimized to allow high quality radiographic projection images while considering both the contrast and resolution as well as manageable scanning times (55 min per sample). The X-ray shadow projections of the three-dimensional (3-D) object were digitized as 2048 × 2048 pixel images and were processed to obtain reconstructed cross-section images using a mathematical algorithm based on the filtered back-projection procedure implemented in the NRecon 1.6.2.0 software (http://www.skyscan.be/products/downloads.htm). This resulted in a 3-D stack of 1500 virtual sections, each consisting of 1500 × 1500 isotropic voxels (where each voxel was 1.7 μm^3^) with a linear X-ray attenuation coefficient, displayed as a grey scale value calibrated between 0 and 255. The entire micro-CT dataset is given as Supporting Information Table S1.

The high resolution tissue scans were conducted at beamline ID19 of the European Synchrotron Radiation Facility (ESRF, Grenoble, France), that is using a parallel-beam geometry of a long (150 m) imaging beamline where the spatial coherence of the beam is particularly large (transverse coherence length in the order of 100 μm). Phase contrast imaging (Cloetens *et al*., 2006) was used for edge detection at cell–cell interfaces. The X-ray beam generated from an 11 pole, variable- gap, high-magnetic field wiggle undulator with a 32 mm magnetic-field period was monochromatized to 19 keV using an artificial multilayer monochromator. The sample-detector distance was set to 90 mm (Weitkamp *et al*., 2010). The image pixel size was 0.75 μm. A total of 1200 projections with an exposure time of 0.1 s was acquired for each sample during a continuous rotation over 180°. The tomographic reconstruction was performed with a filtered backprojection algorithm using the PyHST software, developed at ESRF (Chilingaryan *et al*., 2011), after correction for sample motion using GNU Octave software (http://www.gnu.org/software/octave/). Volume renderings of all samples were obtained by 3-D image segmentation and isosurface representations in Avizo Fire (Visualization Sciences Group, Bordeaux, France). By applying the synchrotron radiation system we could detect objects such as pores somewhat smaller than 1 μm.

### Diffusion modelling

A microscale model of O_2_ diffusion incorporating the real tissue microstructure was used to calculate the O_2_ diffusivities of different regions of rape seed (Ho *et al*., 2011). The model solves the diffusion equations over the 3D tissue geometry consisting of air spaces and cells, to which the known oxygen diffusivity values of air and water are applied. Transport between these compartments was by means of a permeation equation, where the permeability value lumps the permeability of cell wall and cell membrane. The cell wall diffusivity of O_2_ and CO_2_ was assumed to be equal to that of water, while the diffusivity of the cell membrane was used from Ho *et al*. (2011). A numerical experiment was carried out in which O_2_ partial pressure differences of 2 kPa were applied over the microscale geometry and the corresponding fluxes were calculated by means of the microscale model. The apparent diffusion coefficients of the tissues in the microscale sample were calculated from the fluxes and the gas partial pressure difference applied to the boundary of the sample. The diffusivity values of the different types of tissues were calculated. Using the tissue geometry directly avoided the need to use lumped parameters such as porosity and tortuosity to calculate the apparent properties of the tissues.

Three-dimensionally rendered tomographic images of different tissues with edge dimensions of 150 μm were discretized into 100 × 100 × 100 cubical control volumes with an edge of 1.5 μm (after binning). Diffusion model equations (Ho *et al*., 2011; Verboven *et al*., 2012) were discretized over the finite volume grid to yield a linear system of algebraic equations on the unknown concentrations at the nodes. The linear equation system was solved by the conjugate gradient method available in Matlab (The Mathworks, Natick, MA, USA). The program was run on a 16 GB RAM node (Opteron 250; Xeon 5560 and Xeon 5650) of the high-performance computer at VSC K.U. Leuven (Flemish Super Computer Center, Leuven, Belgium).

A numerical experiment was carried out in which O_2_ partial pressure differences of 2 kPa were applied over the microscale geometry and the corresponding fluxes were calculated by means of the microscale model. The apparent diffusion coefficients of the tissues in the microscale sample were calculated from the fluxes and the gas partial pressure difference applied to the boundary of the sample. The diffusivity values of the different types of tissues were calculated.

A macroscale reaction-diffusion model, developed previously (Ho *et al*., 2008, 2011; Verboven *et al*., 2012), was used to predict O_2_ distribution of the intact rapeseed as a result of diffusion and respiration in the developing seed. The 3-D geometry of the entire seed was obtained from the microfocus X-ray CT scans. The seed model had a diameter of 2 mm. The model distinguished the seed coat, hypocotyl, subepidermis layer, cotyledons and endosperm. The distinction of different tissue layers with different properties was accommodated by using the calculated O_2_ diffusivity values obtained from different types of tissue. The spatial variations and anisotropy of the O_2_ diffusivity within each part were neglected. A concentration of 21 kPa O_2_ at 25°C was applied outside the seed. The oxygen consumption was modelled by means of a Michaelis–Menten equation with maximum consumption rates in tissue equal to the measured rate of 0.85 nmol oxygen mg FW^-1^ min^-1^ (as determined in prior tests using whole isolated rapeseed embryos using the method described by Tschiersch *et al*., 2011). To account for differences in the respiratory activity of the three embryo components, we used the flux values derived from a metabolic modelling approach (Borisjuk *et al*., 2013). Accordingly, the relative respiration rate for inner cotyledon, outer cotyledon and hypocotyl was set to 100%, 64% and 54%, respectively. The mitochondrial *K*_m_ value was assumed to be equal to 0.14 μM (1.02 × 10^−2^ kPa; Millar *et al*., 1994). The 3-D model of the rapeseed was developed and solved using the finite volume method in Matlab (Ho *et al*., 2011).

### Measurement of local oxygen concentration and respiration

Oxygen concentration profiles across the seed were measured using needle-type microsensors (for details, see Rolletschek *et al*., 2009). Briefly, oxygen-sensitive optodes (Presens GmbH, Regensburg, Germany) were inserted into the seed using a micromanipulator, and the oxygen concentration was measured at 100 μm intervals along a transect across the seed. Before and after each analysis the sensor was calibrated using premixed gases. For analysing the local respiration rates, we used a fluorescence ratiometric-based device, consisting of an oxygen-sensitive foil and a USB microscope (for details, see Tschiersch *et al*., 2012). Briefly, the seed was cut and the sensor foil was placed on the sample surface. Based on the localized change in fluorescence signal (= oxygen concentration) over time (20 min), information about the localized oxygen consumption can be obtained. In this way, the respiratory activity (in relative units) across the seed was imaged.

### Magnetic resonance imaging (MRI)

MRI experiments for the visualization of both water and lipids were performed on a 17.6 Tesla Avance III (Bruker, Rheinstetten, Germany) wide bore system exactly as described in Neuberger *et al*. (2009).

### Laser microdissection and biochemical analysis

Freeze-dried seeds were used for laser microdissection (for details, see Schiebold *et al*., 2011). After cutting and tissue sampling, the collected embryo material was analyzed for soluble carbohydrates and free amino acids according to the procedures described therein.

### Histological procedures

Histochemical techniques applied to seeds as well as immunostaining were performed as described in Radchuk *et al*. (2012). Immunolocalization was carried out with an affinity-purified anti-cruciferin (Bäumlein *et al*., 2008) polyclonal antibody. Transmission electron microscopy including tissue preparation was performed exactly as described in Borisjuk *et al*. (2002).

## Results

### Extensive airspace is present in the testa and hypocotyl

**Fig 1:**
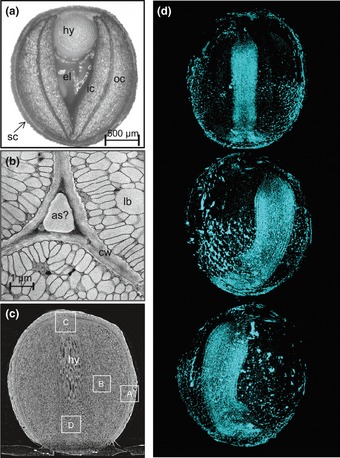
Structural organization of a developing oilseed rape (*Brassica napus*) seed using conventional and non-invasive analysis. (a) Horizontal mid section of a developing seed sampled *c*. 30 d after flowering. (b) A TEM acquired image shows the presence of lipid bodies inside the cotyledonary cells and extracellular voids at the cell corners. (c) A high resolution CT vertical radial section of a whole rapeseed (1.7 μm per pixel). A fuller visualization of these images is provided in Supporting Information Movie S1. The rectangles indicate regions modelled in Figs 2 and 3. (d) A rotation series of views of the void spaces in a seed obtained from high resolution CT. An animated three-dimensional model is given as Movie S2. as, airspace; cw, cell wall; el, endospermal liquid; hy, hypocotyl; lb, lipid body; ic, inner cotyledon; oc, outer cotyledon; sc, seed coat (testa).

**Fig 2:**
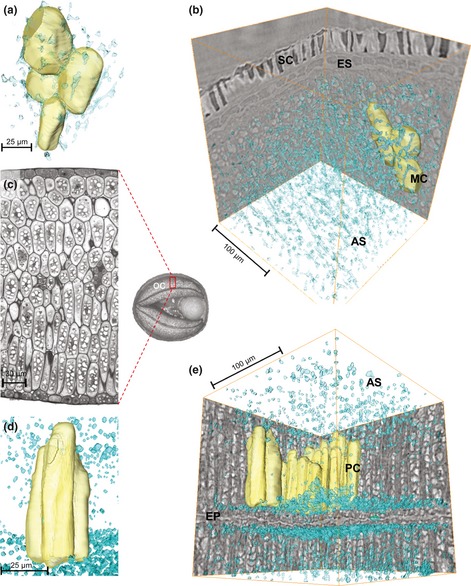
Void spaces in parts of a developing oilseed rape (*Brassica napus*) seed based on synchrotron radiation CT scans (0.75 μm per pixel resolution). (a, b) Three-dimensional model of void spaces and cotyledonary cells facing the testa (region ‘A’ in Fig. 1(c), cfr. vertical radial section). (c) Light microscope image of the outer cotyledon demonstrating the change in cell shape from outer to inner regions. (d, e) Three-dimensional model of void spaces and cotyledonary cells facing the inner cotyledon (region ‘B’ in Fig. 1(c), cfr. vertical radial section). AS, airspace; ES, endosperm; EP, epidermis; MC, mesophyl cells; PC, palisade cell; SC, seed coat (testa).

**Fig 3:**
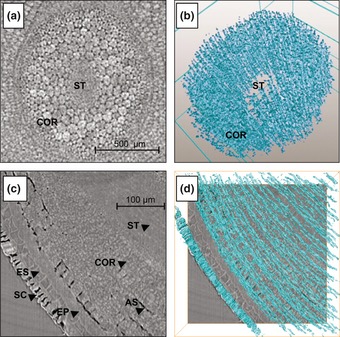
Three-dimensional model of void spaces in the hypocotyl of a developing oilseed rape (*Brassica napus*) seed. (a) CT image of the hypocotyl (corresponding to region ‘D’ in Fig. 1(c), cfr. horizontal section), where the stelar tissue is densely packed. (b) Model of void spaces in a segment of the hypocotyl. Channels in the cortex are aligned axially and are well inter-connected, while in the stele, pores are few in number. (c) CT image of the hypocotyl (corresponding to region ‘C’ in Fig. 1(c), cfr. vertical tangential section). (d) Model of void spaces in the same region described in (c). Here, there are a few longitudinal channels, but their inter-connectivity is poor. AS, airspace; COR, cortex of hypocotyl; ES, endosperm; EP, epidermis; ST, hypocotyl stele; SC, seed coat (testa).

The fully differentiated seed illustrated in Fig. 1(a) was sampled during its prime phase of lipid and protein accumulation. Transmission electron microscopy analysis identified the presence of spaces at the corners of the cotyledonary storage parenchyma cells (Fig. 1b), but was unable to prove either whether they were filled with gas or what their three-dimensional structure was. To visualize the *in vivo* void network of an entire seed, we applied high resolution micro-CT, generating *c*. 1500 virtual sections with an image resolution of 1.7 micron per pixel (Table S1). Fig. 1(c) shows an orthogonal cross through a rapeseed; dark regions in the image indicate air spaces present in the living seed. When the virtual sections were stacked, a high resolution image of the entire seed was obtained (Movie S1), which allowed the voids to be characterized in some detail (Fig. 1d, Movie S2). This analysis demonstrated their diameter to vary from several to a few tens of μm within the testa, and axial channels of diameter up to 5 μm throughout the hypocotyl. However, there was no firm evidence for the existence of voids within the cotyledons, probably because the level of resolution attained was insufficient. As a result, an attempt was made to use high resolution synchrotron radiation micro CT, as this platform provides a roughly twofold improvement in resolution (0.75 μm voxel size). In the cotyledonary tissue facing the testa, small individual voids (diameter 1–5 μm) were present at the corners of the mesophyll cells, but there was no evidence of them being inter-connected (Fig. 2a,b). Further inside the cotyledon, where the cell shape shifted from spherical to columnar (Fig. 2c), there was ample evidence for the presence of voids on the inner surface of the epidermis (Fig. 2d,e), but none were visible between the palisade cells themselves, except in the innermost part of the cotyledon (lower part of Fig. 2e). In the hypocotyl, the (central) stele comprised small, densely packed cells, without any voids (Fig. 3a,b), but the (peripheral) cortex tissue was well endowed with voids, which formed longitudinal channels with a few lateral inter-connections (Fig. 3c,d).

### Modelled levels of porosity, oxygen diffusivity and distribution implied preferential pathways for gas exchange within the seed

The high resolution scans were used to calculate the levels of porosity and oxygen diffusivity in various tissue types. The global diffusion of oxygen gas through the tissue, as represented by its apparent diffusivity, is shown in Table 1. Although the outer epidermal layers of the seed coat have no functional stomata (Geisler & Sack, 2002), the cellular layers of the seed coat in *Brassica napus* are quite porous (14.2%), thanks to the existence of air channels running through it. This enhances gas permeability through the seed coat, and results in a high oxygen diffusivity of on average 60.6 × 10^−9^ m^2^ s^−1^. This value is lower than that expected from the porosity effect only, which is due to the limited connectivity of the pores in the seed coat.

**Table 1:** Porosity and apparent oxygen diffusivity of various tissues of the oilseed rape (*Brassica napus*) seed, calculated using the microscale gas diffusion model

Position	Number of sample	Direction	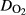 (m^2^ s^−1^) ×10^9^	Porosity (%)
Seed coat	5	–	60.57 ± 69.72	14.2 ± 1.5
Cotyledon sub-epidermis mesophyll	4	–	0.139 ± 0.004	0.72 ± 0.13
Cotyledon mesophyll	4	–	0.149 ± 0.008	0.94 ± 0.05
Cotyledon palisade cells	8	–	0.135 ± 0.001	1.31 ± 0.16
Cotyledon with vascular bundles	4	–	0.136 ± 0.001	0.87 ± 0.10
Hypocotyl	6	Radial	0.184 ± 0.103	1.6 ± 0.1
Hypocotyl	6	Axial	93.17 ± 40.84	1.6 ± 0.1

Values represent means ± SD.

The much higher density of the cotyledonary mesophyll and palisade cell region reduced both the local porosity (to between 0.7% and 1.3%) and the apparent oxygen diffusivity (to 0.135–0.149 × 10^−9^ m^2^ s^−1^), which is obtained from the calculation of oxygen diffusion across the representative 3D tissue sample consisting of pores and cells (see Diffusion modelling in the Materials and Methods section). Porosity in the hypocotyl was about double that in the cotyledons, and the axially aligned voids were well inter-connected. As a result, diffusivity was much higher in the axial than in the radial direction.

**Fig 4:**
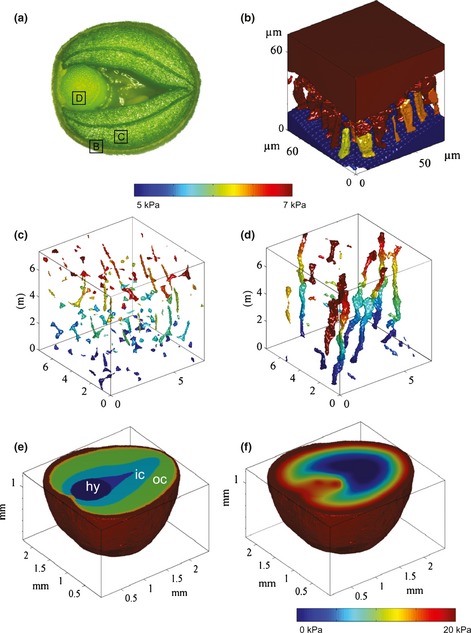
*In silico* modeling of oxygen concentrations in the pore spaces of individual tissues and across the entire oilseed rape (*Brassica napus*) seed. (a) Horizontal cross-section indicating the areas modelled in (b–d): oxygen in (b) the testa, (c) the cotyledonary mesophyll, (d) the hypocotyl; concentrations are colour-coded. At the top and bottom of the computational domain, the oxygen concentrations were set to 7 and 5 kPa, respectively; the lateral sides of the sample were assumed to be impermeable. The *x*, *y* and *z* axis units are 10^−5^ m. (e) Modelled geometry cross-section used in (f); (f) oxygen concentration map (colour-coded) at horizontal cross-section. hy, hypocotyl; ic, inner cotyledon; oc, outer cotyledon.

In the next step, we performed in silico experiments of gas diffusion to calculate both oxygen distribution and exchange rates (Fig. 4). This analysis was based on the whole seed respiration-diffusion model, applying the apparent transport properties of oxygen of the different tissue regions of the seed (Table 1). Oxygen concentrations in the porous part of the testa were high and uniform, given the high localized gas exchange rate (Fig. 4b). By contrast, the poor gas exchange allowed within the less porous cotyledonary mesophyll induced steep oxygen gradients (Fig. 4c,f). In particular, on the inner side of the inner cotyledon, the calculated oxygen concentration was below the *K*_m_ of cytochrome oxidase (Fig. 4f), implying that oxygen diffusion was insufficient to meet the local respiratory demand. Whether this is a realistic picture of what occurs *in planta* depends heavily on the size and shape of the cotyledons and the endosperm, and thus on the developmental stage. The much larger channels present in the hypocotyl should facilitate rapid gas exchange (Fig. 4d), in particular along its axis, so allowing for a high oxygen concentration to develop (Fig. 4f). The modelling also suggested that a degree of radial oxygen loss probably occurs from the hypocotyl, from where it can reach the inner part of the endosperm (Fig. 4f) and beyond to the inner cotyledon. In essence, the variation in porosity and diffusivity within the seed induces major oxygen gradients across the cotyledon; while the oxygen concentration in the centre of the seed is low, it is high in the hypocotyl.

### Modelling of oxygen pool sizes and turnover rates

On the basis of the void structure and the modelling of oxygen distribution therein, we calculated the quantities of oxygen present in the various seed compartments (Table 2). In the seed coat, the amount of oxygen stored in the voids was higher than that stored in the cellular water phase. In the cotyledons, the amount present in the void space was less than that in dissolved form within the cells, whereas in the hypocotyl, a comparable amount was present in both sites. The pore space system as a whole contained *c*. 2.15 nmol oxygen. On the assumption that the seed is fully isolated from its ambient environment, the model estimated that this quantity of oxygen could support only 54 s of mitochondrial respiration (any other O_2_-demanding/producing reaction in seed metabolism was neglected). An additional 0.78 nmol oxygen is held in dissolved form, extending the turnover time to 74 s. Thus the structure of the seed obliges it to replace its oxygen reservoir about once every minute.

**Table 2:** Modelling of oxygen pool size in various oilseed rape (*Brassica napus*) seed compartments, based on micro CT data

	Seed coat	Epidermis	Outer cotyledon	Inner cotyledon	Endosperm	Hypocotyl	Whole seed
Volume fraction	0.22	0.11	0.36	0.20	0.02	0.09	1
Porosity	0.1420	0.0072	0.0104	0.0104	0.0000	0.0160	0.04
Volume (mm^3^)	1.54	0.78	2.57	1.42	0.16	0.67	7.13
Water content (v/v)*	0.81	0.60	0.55	0.60	0.95	0.52	0.63
O_2_ equiv concentration from simulation (mol m^−3^)	8.53	7.95	5.31	2.92	3.26	5.93	
O_2_ pool stored in voids (nmol)	1.860	0.044	0.142	0.043	0.000	0.063	2.153
O_2_ pool dissolved in water (nmol)	0.282	0.115	0.229	0.077	0.015	0.064	0.781
Total O_2_ pool in the seed (nmol)	2.142	0.159	0.371	0.120	0.015	0.127	2.934
O_2_ fraction in tissue	0.73	0.05	0.13	0.04	0.01	0.04	1.00
O_2_ fraction in air	0.87	0.28	0.38	0.36	0.00	0.50	0.73
O_2_ fraction dissolved	0.13	0.72	0.62	0.64	1.00	0.50	0.27

* Data are derived from weighing dissected tissue samples before and after drying at 60°C for 48 h.

### Experimental validation of the modelling approach

**Fig 5:**
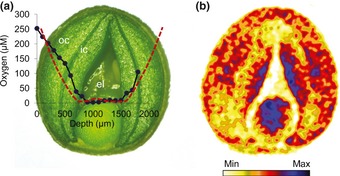
Experimental measurement of oxygen distribution and respiration in the developing oilseed rape (*Brassica napus*) seed. (a) Oxygen concentration profile (blue line) along a horizontal transect through the seed, as determined by a needle-type microsensor. The *x*-axis corresponds to the penetration path of the sensor. The red dashed line indicates modelled oxygen concentrations. (b) Respiratory activity across the seed as determined by a planar oxygen sensor. The resulting respiration map is based on the fluorescence signal of the sensor, where the blue colour indicates peak respiratory activity. el, endospermal liquid; ic, inner cotyledon; oc, outer cotyledon.

*In vivo* measurement of oxygen levels using needle-type microsensors confirmed the presence of a negative gradient from the exterior to the interior of the seed (Fig. 5a). This gradient was slightly steeper in the inner compared with the outer cotyledon. The lowest concentrations (*c*. 3 μM) detected were in the endospermal liquid at the centre of the seed. The agreement between the experimentally determined and the calculated oxygen concentrations was very good (Fig. 5a). Planar oxygen sensors were applied to relate the oxygen concentration profile to the level of cellular respiration. (Such sensors are designed to image oxygen consumption over time, producing data that can be considered to mirror maximum respiratory activity (Tschiersch *et al*., 2012)). A typical characteristic respiration map across a developing seed (Fig. 5b) illustrates that the highest rate of oxygen consumption occurred in the innermost embryonic tissue.

### The hypocotyl is surrounded by liquid endosperm and has a compartmentalized storage metabolism

**Fig 6:**
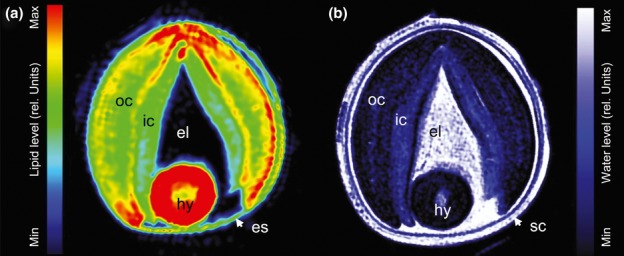
Distribution of (a) storage oils and (b) water in the developing oilseed rape (*Brassica napus*) seed (horizontal section). Visualization was achieved using non-invasive MRI. The concentration of both water and oil are colour-coded and given in relative units. Their three dimensional distribution is given in Movie S3. el, endospermal liquid; es, endosperm; hy, hypocotyl; ic, inner cotyledon; oc, outer cotyledon; sc, seed coat (testa).

Gaseous diffusion is facilitated by inter-connected void spaces, but is much lower when these are filled with liquid rather than with gas (the rate of oxygen diffusion through air is about four orders of magnitude faster than through water). A non-invasive analysis of the three dimensional distribution of both water and storage oils within the developing seed was achieved by the use of magnetic resonance imaging (MRI; Fig. 6). At the mid-storage stage, oils dominated in the hypocotyl and the peripheral parts of the outer cotyledon, while water was accumulated in the testa and endosperm; and slightly elevated water signals were evident for inner vs outer cotyledon. The hypocotyl was surrounded by liquid endosperm. The full 3-D dataset is given as Movie S3.

**Fig 7:**
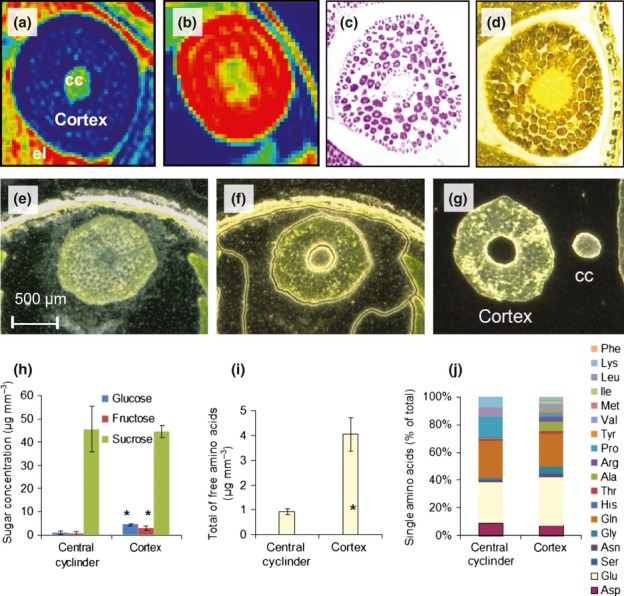
Histological and biochemical analysis of the oilseed rape (*Brassica napus*) hypocotyl. (a) Water and (b) lipid distribution as determined by MRI (low levels marked by blue, high levels by red). (c) Cruciferin distribution, as determined by immunostaining. (d) Starch distribution, as determined by iodine staining. (e–g) For laser micro-dissection procedure. (e) An intact section of the hypocotyl. (f) Tissue regions dissected by laser. (g) Dissected stele and cortex region of the hypocotyl. (h–j) Laser-dissected material used for the analysis of sugars (h) and free amino acids (i, j). *, Statistically significant differences between the cortex and the stele (*t*-test, *P *<* *0.05, *n *=* *6). cc, central cylinder; el, endospermal liquid.

The analysis also detected a radial gradient across the hypocotyl with respect to both water and storage oils, with the highest water and lowest oil levels being present in the stele (Fig. 7a,b). The levels of both the major storage protein cruciferin and starch were particularly low in the stelar cells (Fig. 7c,d). An extended characterization of the metabolism in the hypocotyl was performed using laser-microdissection followed by metabolite analysis (Fig. 7e–j). This showed that the most abundant metabolite was sucrose. The levels of glucose, hexose and total free amino acids were all about threefold higher in the cortex than in the stele. Glutamate (Glu) and glutamine (Gln) were the most abundant free amino acids throughout the hypocotyl, but in the stele there was a strong component of proline (Pro), lysine (Lys) and leucine (Leu). We conclude that storage metabolism and water content were clearly very different in the dense cells of the hypocotyl stele from what occurred in the cortex.

## Discussion

Small seeds, such as those of oilseed rape which are *c*. 2 mm in diameter, may appear homogeneous at the macroscopic scale, and so are commonly assumed to behave as such at the microscopic scale. However, their growth and physiology are actually dependent on several microscopic features, including cell shape, cellular water potential and the presence of intercellular voids. Evidence has been provided here for the existence of voids in the developing oilseed rape seed. Their spatial and geometric complexity has a strong influence on endogenous gas transport and flux, and so may affect local respiratory activity and seed growth.

### The void structure in the developing oilseed rape seed

Gas-filled intercellular voids represent a primary means of within tissue gas transport, given that oxygen diffuses through air so much faster than it can through water. Consequently, tissues that develop a higher proportion of void space will have greater diffusivity than those that do not. Large voids are formed in the oilseed rape developing seed’s testa and hypocotyl, giving them both an ample level of oxygen diffusivity (Table 1) and consequently a high potential for gas exchange. By contrast, the cotyledons (major storage organ of the seed) are poorly endowed with void space (Fig. 2), so gas diffusion in the seed’s interior is restricted. As regards the inter-connectivity of the network, the central stelar region of the hypocotyl lacked voids, while its cortex contained large, well connected voids (Fig. 3). The micro-architecture of the mature root of both the oilseed rape plant (Voesenek *et al*., 1999) and that of other species as well (Verboven *et al*., 2012) similarly comprises a continuous gas space in the cortex but not in the stele, implying that this structure has been laid down already during embryogenesis. The modelling of gas transport in the hypocotyl showed that it is much more effective in the axial than in the radial direction, thereby maintaining the oxygen supply to the radicle immersed in the liquid endosperm (Fig. 6), which contains only a minimal concentration of dissolved oxygen (Figs 4f, 5a). This ensures oxygen supply to the radicle. There is an intriguing parallel here with the response of mature plants to waterlogging stress, where oxygen diffuses within the root cortex downwards to ensure root aeration in the O_2_-depleted soil (Armstrong *et al*., 1994; Verboven *et al*., 2012): if this pathway becomes blocked, then the plant dies. In some plant species, root hypoxia is an important precondition for the formation of voids (so-called aerenchyma) (Armstrong *et al*., 1994; Bailey-Serres & Voesenek, 2008), although this does not occur in oilseed rape (Voesenek *et al*., 1999).

The modelling also suggested a minor component of radial oxygen diffusion from the hypocotyl to the liquid endosperm, from where it can perhaps reach the innermost regions of the oxygen-starved inner cotyledon. In the developing seed, this process is probably therefore beneficial, although in the root, excessive oxygen loss via this route risks hypoxia (but also affords protection from phytotoxins) (Armstrong & Drew, 2002; Colmer, 2003).

The proportion of void space within the cotyledons of the developing seed lies in the region of 1%, and the level of inter-connectivity is poor. By contrast, an extensive void space is required to promote carbon dioxide entry into the mature leaf and to maximize the surface area of the mesophyll in contact with it (Terashima *et al*., 2011; Ho *et al*., 2012). The way in which this void space develops in the leaf is not at all understood, especially given the strength of the current emphasis placed on defining cellular structure (Wuyts *et al*., 2010).

The radial oxygen profiles of the developing seed resemble those characteristic of fruits and tubers, which also have a hypoxic core as a result of impeded oxygen diffusion (Geigenberger *et al*., 2000; Ho *et al*., 2011). The fruit typically avoids anoxia by generating substantial void volume (Ho *et al*., 2011). Unlike the seed hypocotyl, fruits tend not to establish dedicated gas exchange pathways, but rather develop a network of well distributed heterogeneously sized voids of schizogenous or lysigenous origin, which can take up as much as 30% of the fruit’s volume (Verboven *et al*., 2008), a proportion similar to that achieved by the root cortex (Verboven *et al*., 2012). As a result, the rate of gas diffusion is typically orders of magnitude larger than those prevalent in the seed (Ho *et al*., 2011). However, hypoxia could result in physiological disorders and cavity development in fruit during storage (Franck *et al*., 2007).

### Implications of the void structure for respiration and gas exchange in the oilseed rape seed

The developing seed has a high respiratory activity, and can experience oxygen shortage at certain developmental stages and/or under particularly unfavourable environmental conditions (Borisjuk & Rolletschek, 2009). The hypoxic effect on seed composition and growth as well as the metabolic adjustment has been studied intensively (Geigenberger, 2003; Benamar *et al*., 2008; Rolletschek *et al*., 2011), but the level of understanding of the physical constraints over gas diffusion/oxygen supply remains rudimentary. For oilseed rape seeds, the relevance of hypoxia to respiratory activity and storage product synthesis is controversial. While some evidence purports to show that lipid biosynthesis and respiration are enhanced by higher oxygen availability (Vigeolas *et al*., 2003; Musgrave *et al*., 2009), other evidence does not support this (Goffman *et al*., 2005). The respiration maps based on the deployment of oxygen-sensitive foils identified respiratory activity to be at its most intense in the innermost regions of the cotyledon (Fig. 5b). This corresponds to the *in vivo* situation, where photon flux density at the innermost layers of the cotyledon is much lower than further outside the seed, and so this region can generally acquire less energy from photosynthesis (Borisjuk *et al*., 2013). The present modelling data demonstrate that the oxygen concentration in the inner side of the inner cotyledon can fall to levels that limit respiration. Whether or not the supply of oxygen becomes limiting will depend heavily on the size and morphology of both the cotyledon and the endosperm. It seems inevitable that respiratory (and possibly biosynthetic) flux in the interior of the seed suffer from inadequate oxygen supply at particular developmental stages. It has also to be noted that the immediate surroundings of the developing rapeseed (silique atmosphere) can have either higher or lower oxygen concentrations than the standard atmosphere (Porterfield *et al*., 2000; and H. Rolletschek, unpublished data) due to either a release of photosynthetic oxygen by seeds/silique under high light or respiratory consumption during dark periods. The latter might be of special significance because a lower atmospheric oxygen concentration (*c*. 12 kPa in darkness; Porterfield *et al*., 2000) inevitably reduces the driving force for oxygen diffusion into the seed. Our modelling attempt might be used to simulate how this will affect embryo respiration, oxygen diffusion, oxygen distribution within the seed, etc., thereby defining the embryo regions that suffer from hypoxia during the day: night cycle. One also has to keep in mind that the seed’s own photosynthetic capacity may have a significant impact on the embryo′s oxygen status during the light–dark switch, since the level of oxygen generated is at times sufficient to alleviate localized hypoxia (Borisjuk & Rolletschek, 2009).

Although not modelled here, the void structure of the developing seed most likely will affect its exchange capability for carbon dioxide. The concentration of carbon dioxide (largely dissolved as 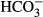 ion) within the oilseed rape seed is some 600 fold greater than in the atmosphere (Goffman *et al*., 2004), mostly due to high rates of fatty acid synthesis (Ruuska *et al*., 2004; Schwender *et al*., 2004). Levels such as these can in general have a significant impact on cellular pH, photosynthesis, respiration and storage activity (Lammertyn *et al*., 2001; Greenway *et al*., 2006; Ho *et al*., 2012). Its effect on enzyme activity may be due to either protein carbamylation and/or a disturbance to reaction equilibria, as has been demonstrated for cytochrome oxidase (Miller & Evans, 1956), succinic dehydrogenase (Cerwick *et al*., 1995) and malic enzyme (Neuberger & Douce, 1980). The carbon dioxide generated will tend diffuse in the direction of its concentration gradient, eventually to be released from the seed via the same physical route as used for oxygen uptake. However, given that the solubility of carbon dioxide in water is so much greater than that of oxygen, the aqueous phase in the seed represents a much lower constraint for diffusion, so that carbon dioxide release is probably less dependent than oxygen uptake on the void structure. Whether this leads to an elevated CO_2_ diffusivity, as was shown for fruits (Ho *et al*., 2006, 2007), remains to be investigated. Distinct diffusivities could, in general, cause distinct gas transport rates, and thus induce a pressure potential that would either need to be alleviated by gaseous nitrogen, or favour permeation, a process that facilitates a higher gas exchange rate than can be attained by diffusion alone (Ho *et al*., 2006).

### Modelling of the seed’s oxygen balance indicates rapid respiration cycles and their promotion by the void space

The modelling has for the first time permitted the estimation of the quantity of oxygen present in either the gaseous or the aqueous phase within the seed and each of the various compartments (Table 2). Relating these estimates to the rate of respiratory oxygen consumption has led to the realization that a developing oilseed rape seed must fully replace its oxygen reservoir about once every minute. This rate may appear surprisingly high, but fits with the previous demonstration that endogenous oxygen concentrations can shift from strongly over-saturated (*c*. 700 μM where photosynthesis occurs under high levels of light) to almost depleted (*c*. 1 μM) within the space of just 3–5 min (Borisjuk & Rolletschek, 2009).

The quantity of oxygen stored in the void space varies between the individual seed compartments, but is globally about threefold higher than the amount present in the aqueous phase. One might therefore conclude that the void structure has some importance as an oxygen storage site (as proposed by Cloetens *et al*., 2006). However, the likelihood is that it is more important for gas exchange, in particular in both testa and hypocotyl due to their high oxygen diffusivity.

Both porosity and oxygen diffusivity are tissue-specific as demonstrated above, and we asked how this is reflected in the pattern of tissue differentiation, including storage activity. For example, the base level of porosity associated with the hypocotyl stele (Fig. 3) is accompanied by a substantial water content and a low level of storage lipid, protein and starch (Fig. 7a–d). Radial oxygen transport from a well aerated root cortex to its stele is inefficient (Table 1), so that any root hypoxia is most likely initially to affect the stele. This feature has been emphasized in root aeration studies (Armstrong & Drew, 2002; Verboven *et al*., 2012). Although possibly coincidental, a distinct functional and evolutionary advantage could be envisioned for the sites of processes that are most limited by internal oxygen depletion (lipid and protein deposition coupled to high respiration) to be located in the peripheral (cortex) tissues of the hypocotyl. For the cotyledon, the major storage organ in the oilseed rape seed, this sort of differentiation, however, was not in evidence. The stele and the cortex also contrasted with respect to their content of both hexoses and free amino acids (the latter containing higher levels of both). Whether this difference reflects distinctive assimilate supply and/or consumption, and whether it has any influence on tissue differentiation or hormone action (Weber *et al*., 2005; Eveland & Jackson, 2011; Sairanen *et al*., 2012) remains an open question, but at any rate, it does serve to underline once more the spatial heterogeneity of the seed. Their tissue-specific analysis at all levels of detail (transcripts, metabolites, etc.) is currently employed in a systems biology approach (Borisjuk *et al*., 2013), and will be a necessity for the better understanding of the functioning and development of a plant seed.

## Supporting Information

Table S1 Micro CT derived data (1500 reconstructed cross-sections) used for the imaging of the void network present in a developing oilseed rape seed (doi: 10.5447/IPK/2012/16)

**Movie S1** Three-dimensional reconstruction of a developing oilseed rape seed based on high-resolution CT images (doi: 10.5447/IPK/2012/15; 444 MB file size). **Movie S2** Three-dimensional model of the void spaces present in a developing oilseed rape seed (doi: 10.5447/IPK/2012/14; 88 MB file size). **Movie S3** Spatial distribution of storage oils (left panel) and water (right panel) in a developing oilseed rape seed.
