# Supplementary material for: Void space inside the developing seed of Brassica napus and the modelling of its function
Source: New Phytol. 2013 May 21;199(4):936–47. doi: 10.1111/nph.12342 (PMC3784975; doi:10.1111/nph.12342)
Supplement: Table S1 — Micro CT derived data (1500 reconstructed cross-sections) used for the imaging of the void network present in a developing oilseed rape seed (doi: 10.5447/IPK/2012/16) [file nph0199-0936-sd1.docx]

**Supporting Information Legends to Table S1 & Movies S1–S3**

**Table S1** Micro CT derived data (1,500 reconstructed cross-sections) used for the imaging of the void network present in a developing oilseed rape seed (DOI: 10.5447/IPK/2012/16 accessible via [http://dx.doi.org](https://exchange.ipk-gatersleben.de/owa/redir.aspx?C=L1Wn7W76d0uh77zqxAH7uUBy8wrqxc8I6KbUzW_NxmpU1o7MLxGhK4GpYAj7eLu5FfWANUy0Og8.&URL=http%3a%2f%2fdx.doi.org))

**Movie S1** Three-dimensional reconstruction of a developing oilseed rape seed based on high-resolution CT images (DOI: 10.5447/IPK/2012/15 accessible via [http://dx.doi.org](https://exchange.ipk-gatersleben.de/owa/redir.aspx?C=L1Wn7W76d0uh77zqxAH7uUBy8wrqxc8I6KbUzW_NxmpU1o7MLxGhK4GpYAj7eLu5FfWANUy0Og8.&URL=http%3a%2f%2fdx.doi.org); 444MB file size).

**Movie S2** Three dimensional model of the void spaces present in a developing oilseed rape seed (DOI: 10.5447/IPK/2012/14 accessible via [http://dx.doi.org](https://exchange.ipk-gatersleben.de/owa/redir.aspx?C=L1Wn7W76d0uh77zqxAH7uUBy8wrqxc8I6KbUzW_NxmpU1o7MLxGhK4GpYAj7eLu5FfWANUy0Og8.&URL=http%3a%2f%2fdx.doi.org); 88MB file size).

**Movie S3** Spatial distribution of storage oils (left panel) and water (right panel) in a developing oilseed rape seed. Signal strengths are color-coded and given in the form of relative units. The *red* bar indicates high oil and the *white* bar high water concentration. The datasets were acquired using MRI.
